# Honey bees of Ethiopia: Their lineages and subspecies based on morphometrics, mitochondrial DNA, and mandibular gland pheromone analyses

**DOI:** 10.1371/journal.pone.0335551

**Published:** 2025-11-07

**Authors:** Walellign Wotro Wanore, Christian W.W. Pirk, Abdullahi A. Yusuf, Steve B. S. Baleba, Mary Chege, Nelly N. Ndungu, Abebe J. Wubie, Workneh Ayalew, Beatrice T. Nganso

**Affiliations:** 1 International Centre of Insect Physiology and Ecology (ICIPE), Nairobi, Kenya; 2 Social Insects Research Group, Department of Zoology and Entomology, University of Pretoria, Pretoria, South Africa; 3 College of Agriculture and Environmental Sciences, Bahir Dar University, Bahir Dar, Ethiopia; University of Alberta, CANADA

## Abstract

Apiculture is a vital economic sector in Ethiopia, providing income and employment for over two million people. However, the classification of the honey bee subspecies in Ethiopia remains debatable. To shed light on this, we analysed wing geometric and classical morphometrics, mandibular gland pheromones, and COI–COII mitochondrial DNA sequences from worker honey bees collected across high, mid and low elevation gradients within Oromia, Amhara, and Southern Nations Nationalities and Peoples’ (SNNP) regions. Our results revealed significant regional morphological and pheromonal variation driven by elevation. Wing size increased with altitude, suggesting adaptive responses to elevation. Classical morphometrics supported this trend, with bees at higher elevation exhibiting larger flight structures. Regional differences in mandibular gland pheromone secretion were also observed, with workers from Amhara secreting the least quantities of these compounds, including the queen substance 9-oxo-2(*E*)-decenoic acid (9-ODA) and its precursor 9-hydroxy-2(*E*)-decenoic acid (9-HDA), as well as the worker component 10-hydroxy-2 (*E*)-decenoic acid (10-HDA) and its precursor 10-hydroxy-decanoic acid (10-HDAA). Furthermore, the secretion of 9-HDA and the total amount of mandibular gland pheromone significantly and negatively correlated with elevation. For mtDNA analysis, all samples from Ethiopia clustered with the Y lineage (*Apis mellifera simensis*) and separated from neighbouring honey bee populations of the A lineage (*A. m. scutellata* and *A. m. monticala*). Overall, our results reveal the significant influence of elevation on adaptive traits of Ethiopian honey bees, which are of the same subspecies.

## Introduction

The Western honey bee (*Apis mellifera* L.) is the most important managed insect pollinator globally, playing a vital role in pollinating both wild flowering plants and agricultural crops [1–3]. Its pollination services are essential for maintaining biodiversity among native plant species, supporting human nutrition, and ensuring food security. Besides pollination, honey bees offer a range of hive products such as honey, propolis, bee venom, beeswax, and pollen, which hold significant value for human health and livelihoods [4–7]. Previous studies reported that *A. mellifera* originated from Africa, Europe, the Middle East, and Asia, and has since spread naturally and/or through globalization of beekeeping to other parts of the world except Antarctica [8–10]. However, recent studies suggested the origin of the honey bees to be North Africa or the Near East based on morphological and molecular datasets [11–13]. Through analyses of morphometric characteristics, mitochondrial DNA (mtDNA), and nuclear markers, approximately 33 subspecies of *A. mellifera* have been delineated [14], belonging to five distinct evolutionary lineages: A (Africa), M (Western and Northern Europe), C (Southern and Eastern Europe), O (From Turkey and the Middle East), and Y (Northeastern Africa) [13,15–17]. However, discrepancies regarding the taxonomy of *A. mellifera* subspecies persist as there are zones of transition where genetic exchange occurs, giving rise to new genotypes or hybrids [14,15]. This is the case in Ethiopia, where several studies have yielded conflicting findings regarding the number of subspecies, their evolutionary lineages, and distribution patterns, underscoring the intricate dynamics of honey bee populations in this country [18–22].

Ethiopia hosts an extraordinary diversity of plant and animal species, owed largely to its varied agroclimatic conditions and vegetation types [23,24]. These encompass a spectrum from Afroalpine to arid and semi-arid, with elevations ranging from as high as 4,533 m above sea level (a.s.l.) in the mountains to as low as 116 m below sea level in the lowlands [23,24]. The diversity of Ethiopian honey bees parallels this variety, with approximately five subspecies reported so far [18–20,25]. However, there have been inconsistencies in these reports. For instance, Smith [18] reported the presence of *A. m. monticola* in the central highlands based on classical morphometric analysis, while Ruttner [25] reported *A. m. scutellata* and *A. m. jemenitica*. Later, Radloff & Hepburn [26] confirmed the presence of *A. m. jemenitica*, *A. m. bandasii*, and *A. m. sudanensis* in the Northern, Central, and Southern parts of Ethiopia, respectively, using morphometric and sting pheromone analyses. Conversely, Amssalu  et al. [20] reported five subspecies across Ethiopia based on morphometric characters which include *A. m. scutellata* in the Southern and Western wet tropical areas, *A. m. jementica* in the Eastern, Northern, and North-western parts, *A. m. monticola* in the Northern highlands, *A. m. bandasii* dominating the Central highlands, and *A. m. weyi-gambella* in the Western and Southern semi-arid to sub-humid lowlands. Meixner et al. [27] reported the occurrence of a unique subspecies *A. m. simensis* of the Y lineage throughout Ethiopia, based on morphometric analysis in comparison to the reference dataset of neighbouring subspecies [17]. Recent genetic and morphometric analyses supported findings by Meixner et al. [27] and further indicated the resemblance of Ethiopian honey bees to neighbouring Eastern African subspecies of the A lineage (*A. m. scutellata* and/or *A. m. monticola*) and Middle Eastern subspecies of the O lineage [21,22]. However, the later studies only sampled honey bees from two of beekeeping regions in Ethiopia: the Tigray (north) and the Southern Nations, Nationalities, and Peoples’ (SNNP) regions [28].

This study, therefore, aimed to reassess the above reports on the diversity and evolutionary lineages of honey bees of Ethiopia, focusing on the three main honey-producing regions: Oromia, Amhara, and SNNP [29,30]. To achieve this, we used four techniques: wing landmark-based geometric morphometric [21,31], classical morphometric [15], mandibular gland pheromones [32,33] and COI-COII mtDNA [17,21,34,35] analyses. All these techniques have been extremely instrumental in deciphering the honey bee subspecies of African origin [26,36–41]. Our integrated approach is expected to provide a comprehensive understanding of honey bee diversity and evolutionary relationships in the country.

## Materials and methods

### Study sites

**Fig 1:**
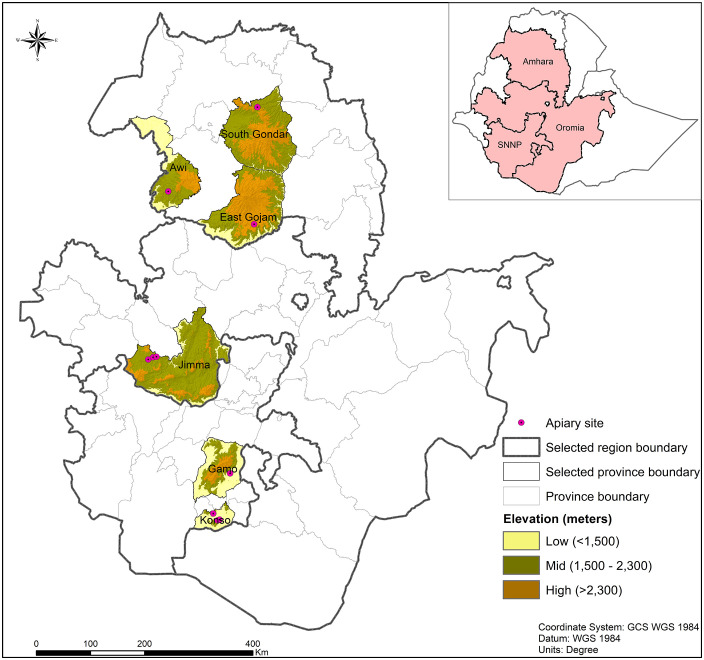
Map showing the location of the six provinces within the Amhara, Oromia, and Southern Nations Nationalities and Peoples regions, and situated within high, mid, and low elevations. The map was created by *icipe* Data Management, Modelling, and Geo-Information (DMMG) unit using QGIS software (https://www.qgis.org/en/site/). The agroecological zones data were sourced from the soil data of Africa (https://africasis.isric.org/#data-catalogue), while the Ethiopia administrative shapefiles were sourced from GADM (https://gadm.org/).

Honey bee samples were collected from Oromia (southwest), Amhara (north and northwest), and SNNP (south) regions of Ethiopia. Honey bee workers were sampled from 31 randomly selected queenright colonies headed by naturally mated queens across 10 apiaries in six provinces (Fig 1). Sampling sites included three apiaries in Konso province, and one apiary in Gamo province, within the SNNP region, three apiaries in Jimma province within the Oromia region; and three apiaries within the Amhara region, each in the Awi, East Gojjam and South Gondar provinces (Fig 1). These apiaries were distributed across the south, southwest, north, and northwest of Ethiopia, and fell within high (>2300 m a.s.l.), mid (1500–2300 m a.s.l.), and low (<1500 m a.s.l.) elevation zones (S1 Table). To ensure spatial independence, these apiaries were located at least five km apart, as honey bees typically forage within three km radius of the hive [42]. 

### Sampling of honey bees

From each of the 31 honey bee colonies used in this study, we randomly collected 60 workers into 50 ml falcon tubes and immediately placed them in an icebox maintained at 0 °C in the field. These worker bees were divided into four groups of fifteen individuals each: one group was used for molecular analysis, another for classical morphometric analysis, a third for molecular analysis, and the fourth for pheromone analysis. Honey bees destined for molecular and morphometric analyses were transferred to cryogenic vials containing 95% ethanol and stored at −80 °C until further analysis, while those for mandibular gland profiling were stored at −20 °C.

### Wing geometric morphometric analysis

**Fig 2:**
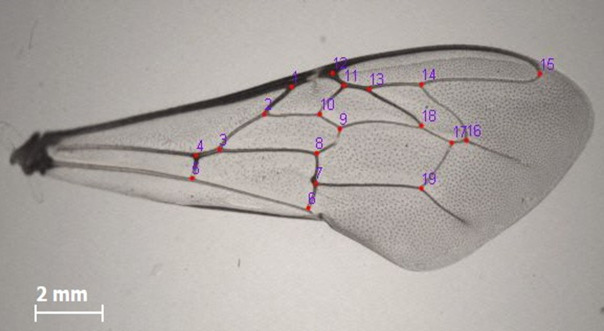
The 19 landmarks on the left anterior wing of *Apis mellifera* used for wing geometric morphometric analyses.

Using sterile forceps, the left anterior wings from each honey bee were dissected and carefully positioned on a microscope slide and covered with a cover slip. Images of the wings were captured using Leica Application Suite v3.4.0 ©2016 software (Leica Microsystems Limited, Switzerland), operated on a Leica EZ4D microscope with a magnification of ×11 [43,44]. Subsequently, the images were converted into TPS files using tpsUtil32 v1.74 and then analysed with tpsDig2 v2.3 [45], where 19 vein intersection points (landmarks) were manually marked on the wings following the protocol of [46] (Fig 2). This was done for 10 worker bees from each colony, and a total of 310 bees were analysed [21,48,50] [21,44,46].

### Classical morphometric analysis

Ten (10) honey bees per colony (310 in total) were used for classical morphometric analysis. Seventeen (17) morphological characters were measured: length of the proboscis (PL), forewing length (FWL), forewing width (FWW), hindwing length (HWL), hindwing width (HWW), length of cover hair on tergite 5 (CHT5), width of wax plate on sternite 3 (WPW), transverse length of wax plate on sternite 3 (WPL), length of sternite 3 (SL), wing angle B4 (B4), wing angle N23 (N23), wing angle O26 (O26), wing angle J16 (J16), length of femur (FL), length of tibia (TL), length of metatarsus (ML) and width of metatarsus (MW) (S1 Fig). Notably, CHT5 length was obtained by averaging measurements taken at five distinct points to represent variability in this parameter: CHT5 (1), CHT5 (2), CHT5 (3), CHT5 (4), and CHT5 (5). The proboscis, left forewing, left hind wing, left hind leg, sternite 3, and tergite 5 were meticulously dissected on a pre-cleaned petri dish, then placed on a microscope slide, covered with another slide, and fixed together with masking tape [46]. Images of the dissected structures were captured using a LEICA-EZ4D microscope with Leica Application Suite version 3.4.0 [Build: 272].

### Extraction and analysis of mandibular gland pheromones of honey bees from Ethiopian

Heads of 10 honey bee workers from each hive were aseptically removed using sterile stainless steel surgical blades, placed individually in pre-labelled 2 ml glass vials containing 200 μl of dichloromethane (ChromSolv® grade for HPLC Sigma-Aldrich, St. Louis, MO, USA) and left for > 24 hours to extract the mandibular gland pheromones. To analyse the mandibular gland pheromones, 100 μl of the dichloromethane extract was transferred into 250 ul glass vial inserts, evaporated to dryness under a gentle stream of charcoal-filtered nitrogen gas, and re-dissolved in 10 μl of an internal standard solution containing 1 mg of octanoic acid and 1 mg of tetradecane in 4 ml dichloromethane and derivatized using 10 μl of bis-(trimethylsily) trifluoroacetamide (BSTFA). One μl of the derivatized extract was then injected into an Agilent 6890 gas chromatograph and analysed according to methods outlined in [33], targeting the following six mandibular gland pheromones: methyl p-hydroxybenzoate (HOB), 9-oxo-2 (*E*)-decenoic acid (9-ODA), 4-hydroxy-3-methoxyphenylethanol (HVA), 9- hydroxy-2 (*E*)-decenoic acid (9-HDA), 10-hydroxy-decanoic acid (10-HDAA) and 10-hydroxy-2 (*E*)-decenoic acid (10-HDA). Identification of each of the components was based on a comparison of their retention times with those of synthetic standards. Quantification was accomplished by comparing the relative mass ratios (RMR) of each of these compounds in a standard solution mixture (containing approximately 1 mg of octanoic acid and 1 mg of tetradecane in 4 mL dichloromethane) to the RMR of tetradecane. We did not separate the enantiomers of 9-HDA in our analysis and reported both together.

### DNA extraction and mtDNA (COI – COII intergenic region) analysis

Genomic DNA was extracted from the thoracic muscles of 31 honey bees, with one worker bee dissected per colony [34,35,47,48], using the ISOLATE II Genomic DNA Kit (BIOLINE, A Meridian Life Science^®^ Company, Germany) following the manufacturer’s protocol. The COI–COII intergenic region was then amplified using the E2 (5’-GGCAGAATAAGTGCATTG-3’) and H2 (5’-CAATATCATTGATGACC-3’) primers [49]. PCR amplification was conducted in a 25 µl reaction volume using MyTaq™ DNA Polymerase (Meridian Bioscience, USA), with cycling conditions consisting of initial denaturation at 95 °C for 2 min, followed by 35 cycles of denaturation at 95 °C for 20 seconds, annealing at 48 °C for 60 seconds, extension at 72 °C for 30 seconds, and a final extension step at 72 °C for 5 minutes. Subsequently, PCR amplicons were visualized on a 1% agarose gel and purified using the ISOLATE II PCR and Gel Purification Kit (BIOLINE, A Meridian Life Science^®^ Company). Bidirectional Sanger sequencing of the purified PCR products was performed at Macrogen Europe. The obtained sequences were then edited, and 30 consensus sequences were successfully generated using MEGA11 software [50]. These sequences underwent BLASTn analysis in the National Centre for Biotechnology Information (NCBI) database and were subsequently deposited in the GenBank database under the accession numbers: PP840172 and PP840205. Alignment of the 30 consensus sequences obtained, along with 5 reference sequences representing three of the honey bee subspecies found in East Africa, *A. m. monticola, A. m. scutellata* and *A. m. simensis* sourced from NCBI (S2 Table), was carried out using ClustalW in MEGA11 version 11.0.13 [50]. The resulting alignment was then used to determine the best substitution model for phylogenetic analysis before constructing a neighbour-joining phylogenetic tree under the Tamura-Nei model with 1000 bootstraps.

### Statistical analyses

Landmark coordinates on the forewings, categorised by sampling sites, were superimposed using full Procrustes fit in MorphoJ software V.1.07 to extract shape and size information from the data and eliminate differences in orientation, position, and isometric size [51,52]. Subsequently, the multivariate analysis of variance (MANOVA) was employed in MorphoJ to determine the variation between sites in terms of wing size and shape, followed by canonical variate analysis coupled with discriminant function analysis to analyse the relative similarities and dissimilarities of wing shape across sites. Spearman’s rank-order correlation analysis was carried out to check the relationship between wing centroid size and elevation, as the elevation data was not normally distributed (Shapiro-Wilk test, p < 0.05) and the variances were not homogenous (Bartlett’s test, p < 0.05). To determine the significance of pairwise differences in mean wing shapes, we performed permutation tests (10,000 rounds) with Mahalanobis and Procrustes distances. The wing centroid size was extracted and further analysed in R-software, using ANOVA, followed by the Student-Newman-Keuls (SNK) *post-hoc* test, to see whether the size of the wings varied among regions and elevation categories since the data met the assumptions of normality (Shapiro-Wilk test, p > 0.05) and homogeneity of variances (Bartlett’s test, p > 0.05).

Measurements of classical morphometric characters in the images were conducted using the ImageJ software package (LOCI, University of Wisconsin). Pixel measurements were converted to mm by calibrating against a mm scale on a ruler captured at the same magnification power (×10 for wings and legs, × 12.5 for proboscis, × 16 for wax plate, and ×25 for cover hairs on the fifth tergite) [46,53]. To compare each classical morphometric character among sites, a KWT followed by *post-hoc* Dunn’s test was performed after confirming that the data for the characters were not normally distributed (Shapiro-Wilk test, p < 0.05) and that the variances were not homogenous (Bartlett’s test, p < 0.05). Spearman’s rank-order correlation analysis was carried out to check the relationship between classical morphometric characters and elevation.

The mandibular gland pheromone profiles were compared among sampling sites using KWT after confirming that the data obtained were not normally distributed (Shapiro-Wilk’s test, *p* < 0.05) and the variances were not homogenous (Bartlett’s test, *p* < 0.05). Spearman’s rank-order correlation analysis was also conducted to assess the relationship between pheromone amounts and elevation. All statistical analyses were performed using R-software V.4.3.2 [54], and colony mean values were used as the unit of replication.

## Result

### Wing geometric morphometric analysis

#### Sizes of left anterior wings from honey bee workers.

**Fig 3:**
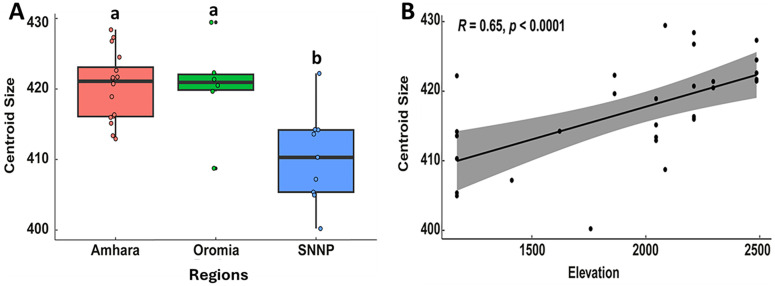
Variation in wing centroid size of the worker honey bees across Ethiopian regions (A), and correlation between centroid size and elevation (B). Boxes illustrate the interquartile range, while lower and upper whiskers indicate the minimum and maximum values, respectively. Different letters on the boxes denote significant differences in wing centroid size among honey bees collected from the three regions, determined by ANOVA followed by the Student-Newman-Keuls (SNK) *post-hoc* Test (*p* < 0.05).

In this study, honey bee populations collected from the three regions in Ethiopia showed significant differences in the size of the left anterior wing centroid (Procrustes ANOVA: F _(2,28)_ = 9.45, *p* < 0.05). Notably, the honey bees in the SNNP region had a smaller centroid size (410.25 ± 2.20) compared to those in the Amhara and Oromia regions (420.25 ± 1.24 and 420.34 ± 2.73, respectively) (Fig 3A). Wing centroid size also varied significantly with elevation (Procrustes ANOVA: F _(2,28)_ = 6.23, *p* < 0.05), with the smallest centroid size observed in the lowland honey bees (411.12 ± 2.32). This result was further supported by a Spearman correlation analysis, which revealed a strong and significant positive association between elevation and wing centroid size (*r* = 0.65, *p* < 0.001) (Fig 3B).

#### Shape of left anterior wings from honey bee workers.

**Fig 4:**
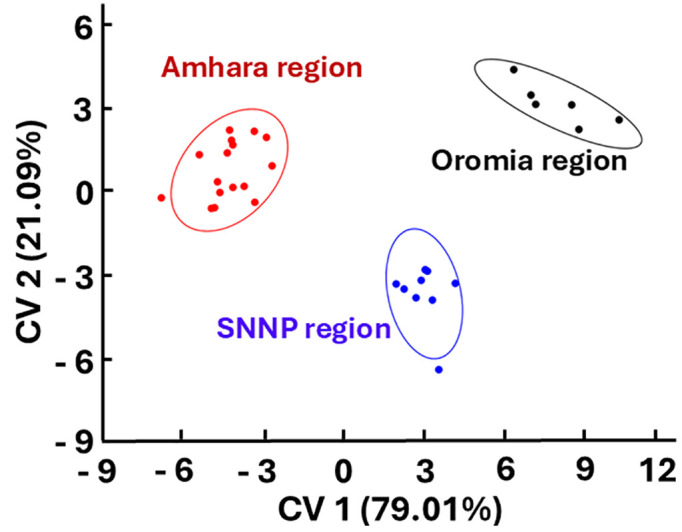
Scatter plot showing the difference in wing shapes of worker honey bees in terms of regions.

The shape of the left anterior wings of honey bee samples collected from the three regions of Ethiopia exhibited significant variation (MANOVA, F _(68,952)_ = 1.74, *p* < 0.05), whereby CV1 and CV2 accounting for 79.01% and 20.09% of the variation, respectively (Fig 4). The results of Mahalanobis distance analysis, which showed significant differences among the three regions, further confirmed this differentiation. In fact, honey bees from the Amhara region showed greater separation from those in the Oromia region (*D* = 12.22; *p* < 0.05), followed by differences between Oromia and SNNP (*D* = 8.41; *p* < 0.05) and between Amhara and SNNP (*D* = 8.38; *p* < 0.05) after 10,000 permutations. However, Procrustes distance analysis revealed significant wing shape differences only between Amhara and SNNP regions (*p* < 0.05), but not between Amhara and Oromia (*p* > 0.05) or Oromia and SNNP regions (*p* > 0.05) after 10,000 permutations. However, the wing shape was not significantly different among workers across elevation gradient (MANOVA, F _(68,952)_ = 1.03 *p* > 0.05), with CV1 and CV2 accounting for 77% and 23% of variation, respectively. None of the pairwise comparisons using Mahalanobis and Procrustes distance analyses were significant (*p* > 0.05).

#### Classical morphometric analysis.

**Fig 5:**
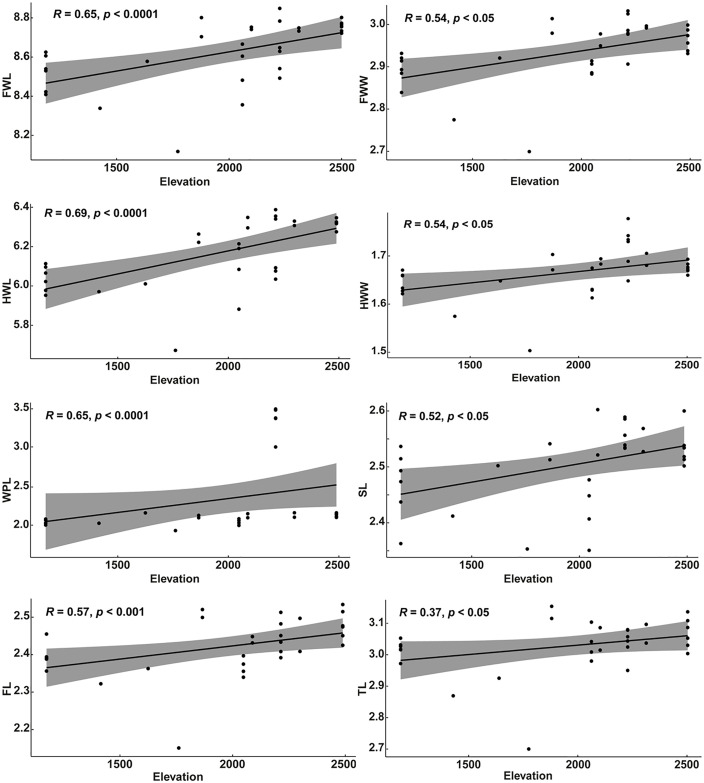
Correlation between elevation and eight classical morphometric characters: the forewing length (FWL) and width (FWW), hindwing length (HWL) and width (HWW), length of wax plate on sternite 3 (WPL), length of sternite 3 (SL), length of femur (FL), and length of tibia (TL).

Among the 17 classical morphological traits examined in this study, nine showed significant differences among the three regions (KWT, *p* < 0.05, S3 Table). These included the forewing length (FWL), forewing width (FWW), hindwing length (HWL), hindwing width (HWW), transverse length of wax plate on sternite 3 (WPL), length of sternite 3 (SL), length of femur (FL), length of tibia (TL), and length of metatarsus (ML). In general, these morphological traits of honey bees from Amhara and Oromia did not differ significantly from each other, but both were significantly different from those of honey bees from the SNNP region. This pattern was consistent with the independent elevation analysis, which showed that several morphological characters, including FWL, FWW, HWL, HWW, WPL and FL, increased significantly with increasing elevation (KWT, *p* < 0.05, S4 Table). Correlation analyses further confirmed weak, moderate or strong relationships between elevation and FWL, FWW, HWL, HWW, WPL, SL, FL, and TL (Fig 5).

#### Mandibular gland pheromones.

**Fig 6:**
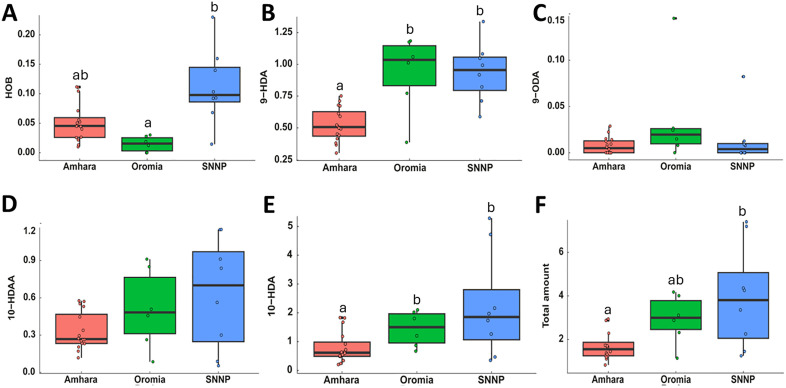
Amounts (mean ± SEM, μg) of mandibular gland pheromones from head extracts of worker honey bees of Ethiopia from the three regions. Boxes with different letters are significantly different from each other (KWT followed by post-hoc Dunn’s test, *p* < 0.05). The five studied mandibular gland pheromone compounds were: HOB = p-hydroxybenzoate **(A)**, 9-HDA = 9-hydroxy-2 (E)-decenoic acid **(B)**, 9-ODA = 9-oxo-2 (E)-decenoic acid **(C)**, 10-HDAA = 10-hydroxy-decanoic acid **(D)**, 10-HDA = 10-hydroxy-2 (E)-decenoic acid (**E**) and the total amount of the five targeted compounds **(F)**. The axis is not in the same scale.

The mean amounts of HOB, 9-HDA, and 10-HDA and the total amounts for all the five compounds found in the mandibular glands of workers (μg) were significantly different among the regions (KWT, *p* < 0.05, Fig 6). Of note, the head extract of the worker compound 10-HDA varied significantly among the honey bee populations of the regions (KWT, H = 7.30, df = 2, *p* < 0.05), with workers from Amhara region exhibiting the least quantity (0.8 ± 0.13 μg) (Fig 6E). Furthermore, the amount of the percussor of the queen substance, 9-HDA, differed among the regions (KWT, H = 14.63, df = 2, *p* < 0.05) (Fig 6C). Elevation has a significant and moderate negative correlation with the secretion of 9-HDA (*R* = −0.53, *p* < 0.05) and a significant but weak negative correlation with the total amount of all the compounds (*R* = −0.39, *p* < 0.05).

#### Analysis of the mtDNA: COI – COII intergenic region.

**Fig 7:**
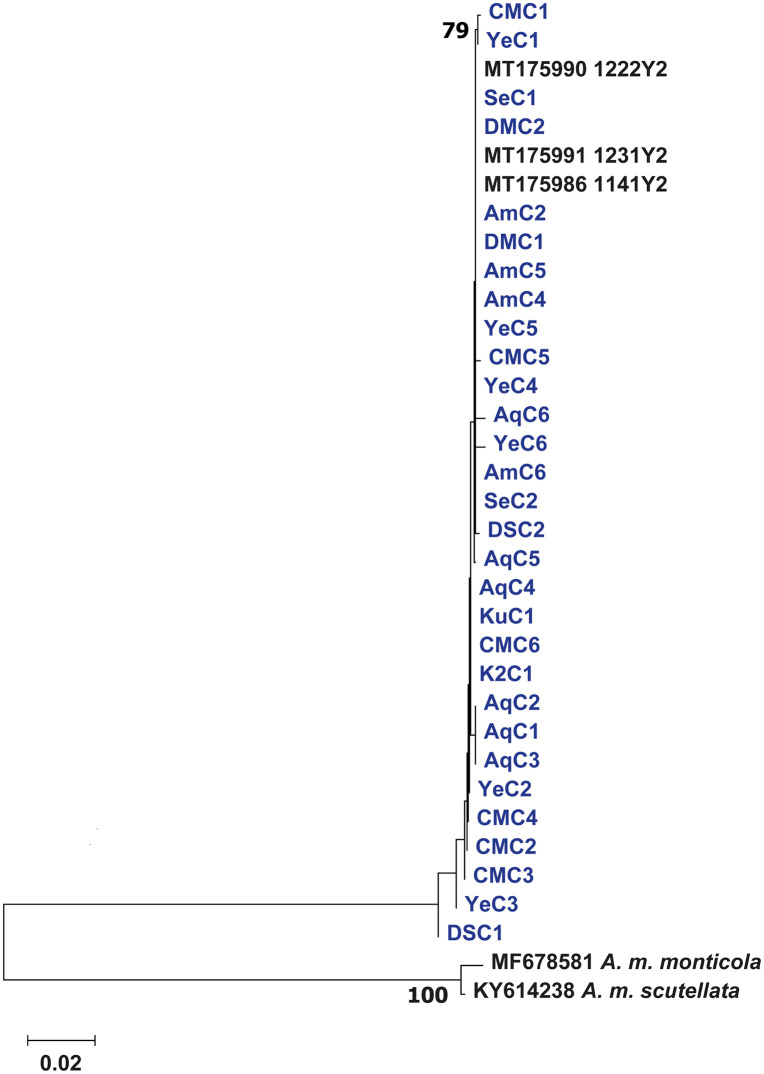
Neighbour Joining phylogenetic tree showing the clustering of the 30 COI – COII consensus mtDNA sequences from the studied honey bee samples collected from Ethiopia (in blue font) among other referenced honey bee sequences from NCBI (in black font). Neighbour Joining phylogenetic tree showing the clustering of the 30 COI – COII consensus mtDNA sequences from the studied honey bee samples collected from Ethiopia (in blue font) among other referenced honey bee sequences from NCBI (in black font). The bootstrap values are indicated by numbers.

In this study, the COI – COII mtDNA sequences of the studied honey bees were successfully sequenced, except the sequence in one colony yielded a mixed sequence chromatogram signal after repeated dilution of the stock solution, thus could not be identified. Analysis of the COI – COII intergenic region through BLASTn in NCBI revealed that out of the 30 consensus sequences obtained from honey bees of Ethiopia, 27 sequences were highly similar to MT175990.1 (99.59–100%), while the remaining three sequences were each highly similar to MT175986.1 (99.87%), MT175991.1 (99.6%), and MT175989.1 (99.6%) (S5 Table). All these sequences belonged to the Y lineage (Y2 haplotype). The Neighbour Joining (NJ) tree based on the alignment of the COI – COII sequences showed that the 30 sequences clustered separately from the two sequences of *A. m. monticola* and *A. m. scutellata* obtained from GenBank, with significant bootstrap support (100) (Fig 7). The samples however did not show any pattern of clustering based on either elevation category or region.

## Discussion

Overall, our study reveals significant morphological and pheromonal variations among honey bee populations of Ethiopia, influenced significantly by elevation gradient. This is expected given the diverse agro-ecologies found across the studied regions of Oromia (southwest), Amhara (north and northwest) and SNNP (south) [55]. Our wing geomorphometric analysis based on the 19 landmarks showed that wing centroid size increases with elevations. This finding was further substantiated by morphometric data indicating an increase in the measurements of various classical morphometric characters along the elevation gradient including the forewing length (FWL) and width (FWW), hindwing length (HWL) and width (HWW), length (WPL) of wax plate on sternite 3, length of sternite 3 (SL), length of femur (FL), and length of tibia (TL) (see Fig 5). In fact, changes in flight structures, particularly wing size and shape, are known to directly influence the lift and aerodynamic forces of insect flight thereby shaping their evolution [56–59]. Relative to the low-elevation flying insects, high-elevation ones such as mountain honey bees [60] and other insect groups [61–63] have evolved longer wings to mitigate aerodynamic challenges brought by significant reductions in temperature, air density and oxygen partial pressure at higher elevations [57]. This suggests that elevation might be the primary driver of morphological differentiation among honey bees of Ethiopia. Taken together, our findings corroborate those of previous studies demonstrating elevation as the dominant factor influencing morphological variation in honey bee populations of Ethiopia [21,27].

The mandibular gland pheromone profiles of honey bee workers of the Y2 haplotype in Ethiopia were often dominated by the worker pheromone 10-HDA and the queen pheromone precursor substance 9-HDA. Ethiopian populations whose profiles were dominated by the worker (10-HDA) and the queen (9-HDA) characteristic compounds resemble subspecies of the northern (*A. m. intermissa*) and northwest (*A. m. sahariensis*) African A lineage [19].

The mitochondrial data provided clear evidence regarding genetic uniformity of Ethiopian honey bee populations with all the samples grouping within the Y lineage (Y2 haplotype); and a clear separation from other subspecies of *A. mellifera* of the A lineage found in the neighbouring countries with strong bootstrap support [12,13] (Fig 7). This finding corroborates the earlier study by Meixner et al. [27], which demonstrated the distinctiveness of the honey bees of Ethiopia from their East African counterparts. Overall, this and previous studies [21,22,27] provide growing evidence that despite notable morphological variation shaped by elevation and agroecological zones, all honey bee populations of Ethiopia belong to a distinct subspecies of the Y lineage, *A. m. simensis*, suggesting a common evolutionary origin. This genetic uniformity across regions is likely maintained by high levels of gene flow, facilitated by the natural bee migration and swarming, both of which are pronounced in African honey bee subspecies [64,65], as well as by human-mediated colony commercialization [22,66], which together limit genetic differentiation despite environmental variation. While the mtDNA is a useful tool for tracing maternal lineages and evolutionary history in honey bees, relying on it alone has a few shortcomings. It masks the genetic share of the paternal line and therefore cannot capture the full genomic complexity, particularly in polyandric species like honey bees. In addition, mtDNA reflects only a single ancestral line and thus provides limited resolution for detecting ongoing hybridization and gene flow [16,46,67].

It is worth mentioning that the presence of the Y lineage of honey bees in Ethiopia is subject to several assumptions of biogeographic phenomena. One explanation suggests that the Y lineage may have migrated across the Sahara during the African Humid Periods approximately 14,800−5,500 years before present, when the Sahara was characterized by savanna vegetation and flowing rivers that supported the movement of humans and animals [68]. Another explanation suggests gradual, stepwise spread facilitated by environmental suitability during the less aridic climate [9]. A further hypothesis attributes the introduction of the Y lineage to ancient beekeeping practices and trans-Sahara trade networks, which could have played a role in its dispersal into Ethiopia [8,15]. Additionally, movement along the Nile corridor has been proposed, as this route has long provided ecological and cultural pathways from central to northeastern Africa, potentially enabling colonization of the Ethiopian highlands [12,15]. Together, these alternative explanations highlight the complex biogeographic history of the Y lineage in Ethiopia.

## Conclusion

Overall, significant morphological and pheromonal differences among honey bee populations from the Amhara, Oromia and SNNP regions of Ethiopia were found in this study, driven by elevation. Elevation-induced increases in wing centroid size suggests adaptations to flight in cooler, low-density air environments. Regional differences in pheromone secretion were observed, with bees from the Amhara region secreting the least quantity of these compounds. The queen pheromone precursor 9-HDA varied across regions and elevation gradient, most likely due to microclimatic or colony-specific effects. The honey bee populations of Ethiopia were found to be genetically distinct from neighboring populations, forming a cohesive group belonging to *A. m. simensis* of the Y lineage. This result underscores the role of elevation in shaping adaptive traits and reinforce the necessity to conserve locally adapted populations to support the sustainability and resilience of the beekeeping sector in Ethiopia.

## Supporting information

S1 Fig Classical morphometric characters showing the body parts of bees where the 17 measurements were taken. (TIF)

S1 Table Names of apiary sites used for honey bee sampling, their provinces, cardinal locations, and elevation. (XLSX)

S2 Table Five reference sequences and their accession numbers belonging to the Cytochrome Oxidase I- Cytochrome oxidase II sub-unit of the mitochondrial DNA of different honey bee sub-species extracted from NCBI database and used for comparative phylogenetic analysis. (XLSX)

S3 Table Comparison (Mean ± SEM) of the 17 classical morphometric characters among the regions. Different superscript letters in each column indicate significant differences among the regions according to Kruskal-Wallis test followed by post-hoc Dunn’s test, p < 0.05. Characters which are significantly different among the apiary sites are highlighted in bold.
(XLSX)

S4 Table Comparison (Mean ± SEM) of the 17 classical morphometric characters among elevation categories. Different superscript letters in each column indicate significant differences among the elevation categories according to Kruskal-Wallis test followed by post-hoc Dunn’s test, p < 0.05. Characters which are significantly different among the apiary sites are highlighted in bold.
(XLSX)

S5 Table Description of the base pair size of COI-COII intergenic region of the 30 consensus sequences, their haplotypes and percent similarity to sequences found in the NCBI nucleotide database (Blastn). (XLSX)

S1 Data Raw data. (ZIP)
