# Supplementary figures and images for: Honey bees of Ethiopia: Their lineages and subspecies based on morphometrics, mitochondrial DNA, and mandibular gland pheromone analyses
Source: PLoS One. 2025 Nov 7;20(11):e0335551. doi: 10.1371/journal.pone.0335551 (PMC12594397; doi:10.1371/journal.pone.0335551)

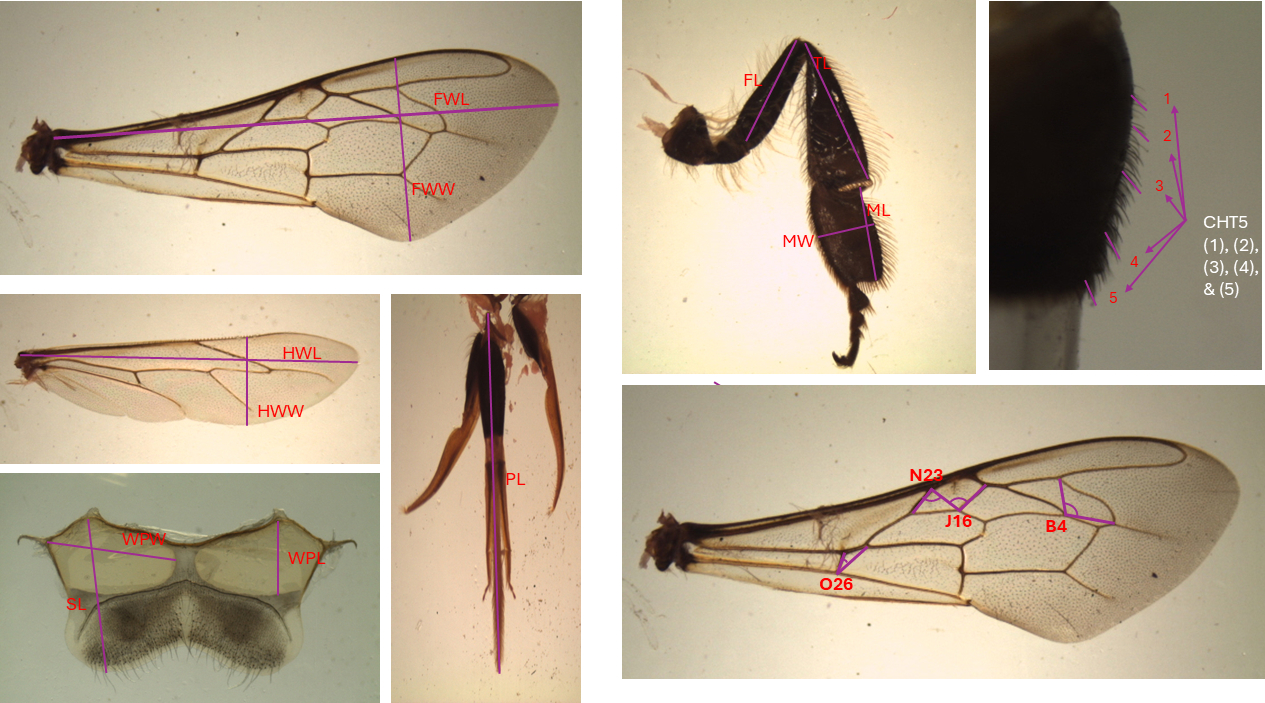

Supplement: S1 Fig — (TIF) [file pone.0335551.s001.tif]
